# Supplementary material for: Chromothripsis during telomere crisis is independent of NHEJ, and consistent with a replicative origin
Source: Genome Res. 2019 May;29(5):737–49. doi: 10.1101/gr.240705.118 (PMC6499312; doi:10.1101/gr.240705.118)
Supplement: Supplemental Material [file supp_gr.240705.118_Supplemental_file_1.zip › contigs/annotated_contigs/DB51/contig.2.DB51_length_337_mean_cov_7.10089020772.docx]

**DB51_length_337_mean_cov_7.10089020772**

```
CTGGTCTTCCCATTCCACACAGCAGCTGTTCCCACCACACCCTCCTCAGATCCCCACCCTTATCCTCATTCC|TTGAGCAAATTA|CTC
 >chr4:30804330-30804402 - E=6e-32 p=3e-02                                           >c
CTTCCATGGCTTATCAAGATTAGCATTCTGCCTTCTGTTTTTACTCTCTATATGCATATATTCACTGGGTATTTCATTTACTTGCATGG
hr4:30802780-30803033 - E=4e-141
AGTCACCTATCACTAAGTGTAAATAAATTGTTTAATTCTTTCCTCCTAATGTTTATCTCCCACATTTCCAATTCCACTTTCTTATTAAA

ATGTTCAAAACTAAAATCTGAAGCAAATATCTTATCCATTACTATTTTCTCTTTTCCCCAACATCTAATCTT
```
